# Subclinical atherosclerosis: what it is, what it means and what we can do about it

**DOI:** 10.1111/j.1742-1241.2008.01804.x

**Published:** 2008-08

**Authors:** P P Toth

**Affiliations:** 1 Sterling Rock Falls ClinicSterling, IL, USA; 2 University of Illinois College of MedicinePeoria, IL, USA

## Abstract

Atherosclerosis is a chronic, progressive, inflammatory disease with a long asymptomatic phase. Disease progression can lead eventually to the occurrence of acute cardiovascular events such as myocardial infarction, unstable angina pectoris and sudden cardiac death. While the disease is still in a subclinical stage, however, the presence of atherosclerosis can be identified by several methods, including coronary angiography, intravascular ultrasonography, B-mode ultrasonography, computed tomography and magnetic resonance imaging. Based on the results of imaging studies, statin therapy can slow, halt or even reverse the progression of atherosclerotic disease, depending on the intensity of treatment. Whether to screen and treat patients for subclinical atherosclerosis remains controversial. Although atheromatous plaque burden reduction has not yet been definitively correlated with significant decreases in risk for acute coronary events in asymptomatic patients, statin therapy contributes significantly to the risk reduction observed in clinical trials in patients with and without overt coronary disease.

**Review Criteria**

Atherosclerotic disease in both its subclinical and clinically established phases is widely prevalent throughout the world. A variety of imaging modalities (B-mode ultrasonography, intravascular ultrasonography, computed tomography, magnetic resonance imaging) have been developed to detect and better define the burden of atherosclerotic lesions in individuals at risk for cardiovascular disease. Statin therapy has been shown to reduce cardiovascular morbidity and mortality in a wide spectrum of patient populations. It is becoming increasingly apparent that statins also impact the course of atherogenesis. The major controlled, randomized studies evaluating the capacity of statin therapy to positively impact the progression of atherosclerotic disease are reviewed herein.

**Message for the Clinic**

Arterial imaging of both the carotid and coronary vasculature can be used to further refine risk based on Framingham risk scoring. Statin therapy favorably impacts rates of atherogenesis. High-dose statin therapy and aggressive low-density lipoprotein cholesterol lowering are associated with atheromatous plaque stabilization and even regression. If subclinical atherosclerotic disease is detected, strong consideration should be given to coupling lifestyle modification with statin therapy, irrespective of overall risk factor burden.

In 2004, coronary heart disease (CHD) affected an estimated 15.8 million people in the USA and was responsible for one of every five deaths, making it the single largest cause of mortality in American men and women (1). In the UK, CHD causes over 105,000 deaths annually; approximately 21% of deaths in men and 15% of deaths in women (2). The prevalence of CHD increases with age, particularly after age 55 years in men and age 65 years in women, although many younger adults experience acute coronary events secondary to the development of premature CHD. Each year approximately 700,000 Americans have their first coronary event, and 500,000 have a recurrent event. Those who survive the incident event have substantially higher risk of recurrent coronary events and sudden death, as well as heart failure and stroke. In addition to increasing morbidity and mortality, CHD places a substantial economic burden on society, with total annual costs in the USA estimated at $151.6 billion (1). This total includes $83.6 billion in direct medical expenditures and $68.0 billion in lost productivity because of CHD morbidity and premature mortality. When other types of cardiovascular disease are included, the prevalence rises to 79.4 million people and the total annual cost to $431.8 billion (1). Similarly, CHD costs the healthcare system in the UK around £3500 million annually. Hospital care for people who have CHD accounts for about 79% of these costs, while buying and dispensing drugs accounts for a further 16% (2).

Coronary heart disease is a clinical manifestation of atherosclerosis, a chronic and progressive inflammatory disease. Atherosclerosis begins early in life. Postmortem evaluation of hearts of young (mean age, 22.1 years) men killed during the Vietnam conflict demonstrated that almost 50% had some evidence of coronary atherosclerosis (3). Similarly, postmortem analysis of coronary vessels from teenagers and young adults who died from other causes showed evidence of early atherosclerotic lesions and, in some cases, fibrous plaques (4,5). In the latter two studies, the extent of coronary atherosclerosis was directly associated with ante-mortem concentrations of low-density lipoprotein cholesterol (LDL-C) and negatively associated with high-density lipoprotein cholesterol (HDL-C). Although subclinical vascular disease is asymptomatic, it can lead to both physical and cognitive dysfunction if left untreated (6).

## Atherogenesis

Atherogenesis begins at sites of endothelial injury. Such injury may result from a variety of factors, including increased local shear forces from hypertension, elevated plasma concentrations of LDL-C and remnant lipoprotein particles, chemical toxins in cigarette smoke, low serum HDL-C and impaired reverse cholesterol transport, insulin resistance, and glycosylated end product formation in diabetes mellitus, among others (Table 1) (7–9). These factors decrease endothelial cell production of nitric oxide, thereby impairing vasodilatory capacity and the normal barrier and protective functions of the vascular endothelium. As a result, LDL-C infiltrates the subendothelial space, where it can be oxidatively modified by a variety of enzymes (e.g. 5′-lipoxygenase, phospholipase A2 and myeloperoxidase) (10). Dysfunctional endothelial cells express a number of adhesion molecules (vascular cell adhesion molecule-1, intercellular adhesion molecule-1) and selectins that promote the binding of circulating monocytes to vascular endothelial cells (10). Once attached, monocytes are exposed to monocyte chemoattractant protein-1, a chemokine that promotes the transmigration of bound monocytes into the subendothelial space. There is evidence that monocytes can access the vessel wall via direct endothelial transcytosis or between endothelial cells with impaired gap junctional association (11). The production of monocyte colony stimulating factor in the inflamed intima promotes the differentiation of monocytes into macrophages. Macrophages exposed to modified LDL express scavenger receptors (SRA, CD-36) that bind and promote the internalisation of oxidised LDL and a broad range of other particles and cell fragments (8,10). As the macrophage progressively accumulates more and more cholesterol, cytosolic lipid droplets form, and the macrophage takes on the appearance of a lipid-laden foam cell.

**Table 1:** Risk factors contributing to atherosclerosis

Genetics (family history)
Diabetes mellitus
Obesity
Cigarette smoking
High serum levels of low-density lipoprotein cholesterol
Low serum levels of high-density lipoprotein cholesterol
Physical inactivity
Hypertension
Age (> 55 years in men, > 65 years in women)
Systemic inflammatory states

T-lymphocytes and other mediators of inflammation and immunity infiltrate the developing lesion site from both the intimal and adventitial aspects of the vessel wall (12). Once localised to the various layers of the arterial wall, T cells can secrete inflammatory cytokines and growth factors, which in turn provide a signal for smooth muscle cells to alter their cytoskeleton, produce matrix metalloproteinases, and migrate from the media into the intimal space, where they proliferate and secrete extracellular matrix components that form a fibrous cap over the developing lesion (8,10,13).

The cytokines released as part of the inflammatory cascade stimulate production of interleukin-6, which in turn stimulates the production of C-reactive protein (CRP) and other acute-phase reactants (10). It is important to recognise that the ongoing inflammatory response in the vascular wall continues to provide signals for further LDL uptake and leucocyte infiltration, creating conditions for further growth of the atherosclerotic lesion (9). Over time, the atherosclerotic lesion continues to expand at its base via the same mechanisms that led to formation of the initial fatty streak.

The stability of the advanced atherosclerotic lesion or plaque depends on its cellular and extracellular contents. Plaques with small lipid cores, thick fibrous caps, few inflammatory cells and a preponderance of smooth muscle cells are typically stable; conversely, those with large lipid cores, thin fibrous caps, numerous macrophages and relatively few smooth muscle cells are most likely to rupture (9,14). Activated macrophages in the developing plaque secrete matrix metalloproteinases that degrade and weaken the matrix component of the fibrous cap. They also secrete various cytokines that inhibit smooth muscle proliferation and matrix production and promote smooth muscle cell apoptosis (14). The inflammatory process can be kept in balance by the actions of smooth muscle cells, which provide the matrix components necessary to strengthen and stabilise the fibrous cap.

Plaque rupture usually occurs at the base of the fibrous cap, which is usually thin and contains relatively few smooth muscle cells and many inflammatory cells. When the plaque ruptures, platelets and coagulation factors in circulating blood are exposed to the thrombogenic contents of the plaque’s extracellular matrix and lipid core, including collagen, phospholipids and tissue factor (10,13). Thereafter, the disrupted plaque serves as a scaffold to allow platelet aggregation and coagulation. The formation of thrombin, subsequent conversion of fibrinogen to fibrin, and release of von Willebrand factor from activated platelets creates a cross-linked network that allows a thrombus to form. The thrombus size depends on the extent of plaque rupture as well as activity of the endogenous fibrinolytic pathway. When sufficiently large, the thrombus can either partially or completely occlude the coronary vessel lumen and precipitate an acute coronary event (9).

## Atheromatous plaque burden

Many plaque ruptures are clinically silent but can be detected on postmortem assessment as breaks in the fibrous cap associated with evidence of healing (15). In a cohort of 142 men who died of sudden coronary death, 86 hearts (61%) had evidence of healed silent ruptures (15). Notably, however, acute plaque rupture occurred commonly in arteries with evidence of previously healed silent ruptures that demonstrated greater luminal narrowing than in arteries without these healed ruptures. Moreover, arterial luminal narrowing increased with the numbers of healed sites of previous plaque rupture. Immuno-histochemical analysis revealed that smooth muscle proliferation was increased at healed rupture sites, suggesting that the occurrence and healing of silent plaque ruptures contributes to further expansion of atherosclerotic plaque (15).

The prevalence and extent of atherosclerosis increase with age. In the Framingham risk assessment, age is used as a surrogate for coronary plaque burden, but plaque burden is the true risk factor for CHD-related morbidity and mortality (16). Because plaque burden can vary among individuals at any given age, accurate measurement of subclinical atherosclerosis may provide a better method for predicting risk for acute cardiovascular events.

Markers of inflammation are regarded as surrogates of asymptomatic atherosclerosis, inasmuch as inflammation plays a central role in the atherogenic process. Higher CRP levels have been consistently associated with increased risk for major cardiovascular events across numerous populations and have also been shown to add prognostic information to LDL-C levels and Framingham risk scores (17). However, the role of CRP as an aetiologic factor in atherosclerosis is not yet clear (18). In the Dallas Heart Study, a population-based cohort of nearly 3400 subjects aged 30–65 years (19), higher CRP levels were associated with a modest increase in the prevalence of subclinical atherosclerosis as detected by electron-beam computed tomography (EBCT) or magnetic resonance imaging (MRI), but this association was not independent of traditional cardiovascular risk factors. The authors concluded that CRP is a poor predictor of atherosclerosis disease burden. In the Inflammation and Carotid Artery – Risk for Atherosclerosis Study (20), higher CRP levels were associated with morphologic features of progressive carotid atherosclerosis in asymptomatic CHD patients. Whether or not CRP proves to be causally related to the atherothrombotic process, it appears to be a sensitive biomarker for quantification of cardiovascular risk (21). The Justification for the Use of statins in Primary prevention: an International Trial Evaluating Rosuvastatin (JUPITER) (22), was designed to determine whether statin therapy reduces vascular event rates in persons with normal levels of LDL-C and elevated levels of CRP. In an interim analysis by this study’s Data Safety and Monitoring Board, the JUPITER trial has been terminated earlier than expected because rosuvastatin therapy demonstrated significant capacity to reduce both mortality and cardiovascular morbidity compared to placebo.

## Prevalence of subclinical atherosclerosis

Although the exact prevalence of subclinical atherosclerosis is undetermined, it is noteworthy that 50% of men and 64% of women in the USA who die suddenly from CHD have no prior manifestation of disease, and the majority of these individuals were not considered to be at high risk according to Framingham risk stratification. In an assessment of more than 5000 adults aged ≥ 65 years participating in the Cardiovascular Health Study, the prevalence of subclinical atherosclerotic disease was 36% in women and 38.7% in men and increased with age (23). In a later study, 318 asymptomatic subjects were randomly sampled from the Framingham Offspring Study cohort based on strata of sex, age and Framingham Risk Score. Cardiovascular MRI revealed that 38% of women and 41% of men had evidence of aortic atherosclerosis, and, again, plaque burden increased with age group (24).

A plethora of studies have found increased prevalence of subclinical atherosclerosis as measured by CT and/or carotid ultrasound in discrete population groups without evidence of CHD, including postmenopausal women (25), women with a family history of premature CHD (26), and European Americans as compared with African Americans (27), among others. It is apparent that metabolic disorders contribute to subclinical atherosclerosis, which has been identified in persons with impaired fasting glucose (28), persons with the metabolic syndrome (29–31), and those with diabetes mellitus (31–33).

## Techniques for measuring subclinical atherosclerosis

A variety of invasive and non-invasive techniques are available to measure atherosclerosis and subclinical atherosclerosis (Table 2). These techniques can ascertain parameters such as luminal diameter or stenosis, vessel wall thickness, plaque volume, and the specific distribution and localisation of atherosclerotic disease. Accordingly, although atherosclerosis may be asymptomatic, it can be identified and quantified within specific distributions of the vasculature.

**Table 2:** Comparison of techniques for identification of subclinical atherosclerosis

**Characteristic**	**Coronary angiography**	**Intravascular ultrasound**	**B-mode ultrasound**	**Magnetic resonance imaging**	**Electron-beam computed tomography**
Invasive	Yes	Yes	No	No	No
Primary measure	Stenosis	Plaque volumeand composition	Intimal-medialthickness	Plaque volumeand composition	Coronary arterycalcification
Plaque composition	No	Yes	No	Yes	No
Plaque burden	No	Yes	No	Yes	Yes
Plaque vulnerability	No	Yes	No	Yes	No

### Non-invasive techniques

B-mode ultrasonography can determine the combined thickness of the arterial intimal and medial layers, usually measured in the common carotid artery (34). The carotid intima-media thickness (IMT) reflects the diffuse thickening of the intimal layer seen in atherosclerosis and has been validated as a measure of the risk for cardiovascular events (35) and atherosclerotic disease burden (36). High-resolution MRI non-invasively evaluates plaque volume and composition, fibrous cap integrity, and lesion type. Consequently, it provides a measure of both plaque burden and susceptibility to rupture (37,38). Both B-mode ultrasound and high-resolution MRI are currently used only in clinical trial settings and are not yet developed enough for individual patient diagnostics. It remains to be established how reproducible IMT measurements are outside of core laboratory settings.

Electron-beam CT, which is used clinically, measures coronary artery calcification. Coronary calcium reflects plaque burden, because calcium deposits are related to the lipid and apoptotic remnants of the plaque (39). Coronary artery calcification is an independent cardiovascular risk factor (40) that adds prognostic information when considered in conjunction with other risk factors (41) and when used in patients with a 10-year CHD risk of > 10% based on Framingham risk scoring (42). However, although EBCT can localise coronary plaques within the coronary tree and provide a quantitative measure of relative disease severity, it cannot be used to ascertain the susceptibility of individual plaques to rupture.

Contrast-enhanced multislice CT, also known as MDCT, uses electrocardiogram-gated images to quantify atherosclerotic calcification in the coronary arteries (43,44). Because of its enhanced sensitivity, it is generally used for detailed studies of the coronary anatomy, whereas EBCT, which uses relatively little radiation, is more often reserved for studies quantifying coronary artery calcification.

### Invasive techniques

Coronary angiography localises plaque and reveals degrees of coronary luminal stenosis (as such, it provides a ‘lumenogram’). Although the presence and number of high-grade stenoses are associated with increased risk of future coronary events, angiography cannot identify culprit lesions that are prone to rupture and cause acute coronary syndromes (ACS) (45). Intravascular ultrasound (IVUS), another experimental technique, is an invasive procedure made possible by the miniaturisation of ultrasound transducers that can be placed at the tip of a coronary catheter (46). Unlike coronary angiography, which identifies the impact of atherosclerosis on the vessel lumen, IVUS can quantify the size and composition of plaques along the entire thickness of the vessel wall and, consequently, provides information about lesion location and the magnitude of plaque burden. IVUS can also identify ectatic plaque that is not yet infringing on the arterial lumen. IVUS is not a screening tool, however, and is currently used only as an experimental technique.

The promise of these techniques is that a single measurement can provide valuable information about the distribution and localisation of subclinical atherosclerosis. Serial measurements made over time provide the opportunity to evaluate the impact of risk factor treatment on atherosclerotic disease progression and, perhaps, changes in risk for ACS.

## Predictive value of cardiovascular imaging techniques

To determine whether carotid IMT improved prediction of coronary events compared with the Framingham Risk Score, Baldassarre et al. (47) studied 1969 dyslipidaemic patients considered to be at low or intermediate risk who underwent carotid ultrasound at a lipid clinic. Both the Framingham Risk Score and the carotid IMT were independent predictors of outcome (p < 0.04 for both), and the IMT significantly improved the predictive value of the Framingham Risk Score (p = 0.04). Patients whose Framingham scores placed them at intermediate risk were determined to be at high risk when their carotid IMT was above the 60th percentile (men) or the 80th percentile (women) of the maximum IMT distribution.

Two recently published meta-analyses were conducted to determine the extent to which surrogate markers for atherosclerosis predict future cardiovascular end-points. Simon et al. (48) used published data of prospective studies of subclinical disease to calculate the incidence of cardiovascular end-points associated with subclinical atherosclerosis as identified by carotid IMT, carotid ultrasound, elevated coronary artery calcium on CT examination, decreased ankle–arm index pressure assessed by Doppler, or pulse wave velocity assessed by mecanography. They found that while the yearly incidence of coronary events was < 1% in the absence of atherosclerosis, it increased to > 1–3% in the presence of atherosclerosis, depending on the marker tested. The authors concluded that detection of subclinical, asymptomatic disease is a worthwhile screening test for predicting future cardiovascular events (48).

Lorenz et al. (35) performed a systematic review and meta-analysis of eight studies examining the association between carotid IMT and vascular events. Using random effects models, they found that adjusted for age and sex, the relative risk of a myocardial infarction per one standard deviation difference in common carotid artery IMT was 1.26 [95% confidence interval (CI): 1.21–1.30] and per 0.10-mm difference it was 1.15 (95% CI: 1.12–1.17). The relative risk for stroke per one standard deviation difference in the common carotid artery IMT was 1.32 (95% CI: 1.27–1.38), and per 0.10-mm difference it was 1.18 (95% CI: 1.16–1.21). Age distribution, carotid segment definition and protocol for measuring IMT represented sources of heterogeneity among the studies. Based on this meta-analysis, the authors concluded that carotid IMT is a strong and valid predictor of vascular events (35).

## Prevention and treatment of subclinical atherosclerosis

Atherosclerosis is a chronic and progressive disease whose optimal prevention requires lifelong attention to diet and exercise, smoking abstinence, and aggressive risk factor identification and treatment. National and international treatment guidelines recognise the importance of LDL-C in driving atherogenesis and CHD risk and recommend stratified target LDL-C levels for reducing this risk. When diet and exercise alone are not sufficient for achieving the LDL-C goal, statin therapy is most frequently initiated. Numerous studies have shown that statins not only reduce CHD morbidity and mortality but also decrease the rate of atherosclerotic disease progression and, under some circumstances, can even induce the regression of atherosclerotic plaque (36,49–53).

### Patients With CHD

In studies using B-mode ultrasonography in patients with CHD, statin therapy slows the rate of atherosclerosis progression in the carotid artery. In an early study, pravastatin 40 mg/day reduced progression of atherosclerosis by 35% in the common carotid artery over a 3-year period; the carotid IMT increased by a mean of 0.029 mm/year with pravastatin compared with 0.046 mm/year with placebo (p = 0.03) (49). However, pravastatin did not significantly affect the change in IMT at the carotid bifurcation or internal carotid artery. In a more recent study, intensive therapy with atorvastatin 80 mg/day, which resulted in a mean LDL-C of 1.97 mmol/l (76 mg/dl), reduced carotid IMT by 0.034 mm over 12 months, whereas the carotid IMT increased by 0.025 mm in the group assigned to moderate lipid lowering with pravastatin 40 mg/day [achieved LDL-C, 2.84 mmol/l (110 mg/dl)] (36).

Studies using IVUS imaging have yielded more dramatic results, and the clinical benefits were related, once again, to the intensity of statin therapy. The Reversal of Atherosclerosis with Aggressive Lipid Lowering trial (50) compared moderate lipid lowering with pravastatin 40 mg and intensive lipid lowering with atorvastatin 80 mg in 654 patients with angiographic evidence of luminal narrowing ≥ 20% in at least one coronary vessel. LDL-C was lowered to a mean of 2.84 mmol/l (110 mg/dl) with pravastatin and 2.04 mmol/l (79 mg/dl) with atorvastatin. Intensive therapy with atorvastatin prevented progression of coronary atherosclerosis; the atheroma volume was essentially unchanged (−0.4%) over the 18-month follow-up, whereas it increased by 2.7% in the pravastatin group (p = 0.02) (50). In A Study to Evaluate the Effect of Rosuvastatin on Intravascular Ultrasound-Derived Coronary Atheroma Burden (51), intensive therapy with rosuvastatin 40 mg/day, which achieved a mean LDL-C of 1.58 mmol/l (61 mg/dl), significantly reduced the percent atheroma volume by 0.98% compared with baseline over 2 years (p < 0.001). For the secondary end-point of median change in the most diseased 10-mm subsegment of a target coronary vessel, plaque volume decreased by 5.6% (p < 0.001). This finding suggests that intensive statin therapy designed to achieve very low LDL-C levels can in fact reverse coronary atherosclerosis to some degree.

### Patients with subclinical atherosclerosis

Aggressive statin therapy reduces carotid IMT in patients with familial hypercholesterolaemia, a population at high risk of premature CHD. In the 2-year Atorvastatin versus Simvastatin on Atherosclerosis Progression trial (52), a randomised, controlled trial of 325 patients with familial hypercholesterolaemia, atorvastatin 80 mg/day reduced the mean carotid IMT from baseline by 0.031 mm; this differed significantly from the mean 0.036-mm increase seen with simvastatin 40 mg/day (p = 0.0005). In the atorvastatin group, mean decreases in IMT from baseline were observed in the common carotid artery (−0.041 mm, p = 0.001) and internal carotid artery (−0.032 mm, p = 0.03), but not at the carotid bulb (−0.022 mm, p = 0.37) (52). The impact of aggressive LDL-C lowering on carotid IMT was explored in the Ezetimibe and Simvastatin in Hypercholesterolemia Enhances Atherosclerosis Regression trial (54). In this 2-year study, 725 patients with familial hypercholesterolaemia were randomly allocated to treatment with simvastatin 80 mg plus ezetimibe 10 mg or to simvastatin 80 mg alone. Preliminary results announced in January 2008 showed that, although there was a significantly greater reduction in LDL-C with combination therapy vs. simvastatin alone (58% vs. 41%; p < 0.01), this reduction was not associated with a difference in slowing atherosclerosis progression over 2 years: carotid IMT increased by 0.0111 mm in the combination therapy group and by 0.0058 mm in the monotherapy group (p = 0.29) (55).

Aggressive statin therapy with rosuvastatin was recently shown to arrest atherosclerotic disease progression. The Measuring Effects on Intima-Media Thickness: an Evaluation of Rosuvastatin (METEOR) study (53) enrolled 984 persons with age as the only CHD risk factor or a 10-year Framingham Risk Score < 10%, modest carotid IMT thickening (1.2–3.5 mm), and elevated LDL-C [mean, 3.98 mmol/l (154 mg/dl)]. Subjects were randomly assigned to rosuvastatin 40 mg/day or placebo for 2 years. LDL-C was reduced to a mean of 2.02 mmol/l (78 mg/dl) in the rosuvastatin group and was essentially unchanged in the placebo group. Over the 2-year study period, rosuvastatin slowed the progression of maximum carotid IMT, measured by B-mode ultrasound at 12 carotid sites, compared with placebo [−0.0014 mm/year (95% CI: −0.0041 to +0.0014) vs. +0.0131 mm/year (0.0087–0.0174), p < 0.001]. Comparable results favouring rosuvastatin were seen when maximum IMT was measured in the common carotid artery (−0.0038 vs. +0.0084 mm/year, p < 0.001), carotid bulb (−0.0040 vs. +0.172 mm/year, p < 0.001) and internal carotid artery (+0.0039 vs. +0.0145 mm/year, p = 0.02) (53). Thus, METEOR showed that in a low-risk population with evidence of subclinical atherosclerosis, rosuvastatin either significantly reversed (in common carotid artery) or stopped or significantly arrested (in the other segments) the progression of atherosclerosis. Based on the results of METEOR, the US Food and Drug Administration in 2007 approved an indication for the use of rosuvastatin as an adjunct to diet in slowing the progression of atherosclerosis (56).

The effect of conservative vs. aggressive statin treatment on carotid atherosclerosis was also evaluated by high-resolution MRI (57). A total of 43 subjects with evidence of carotid stenosis measured by ultrasound or evidence of a plaque with a lipid-rich necrotic core identified by MRI were enrolled in the Outcome of Rosuvastatin Treatment on Carotid Artery Atheroma: a Magnetic Resonance Imaging Observation (57) and were treated with rosuvastatin 5 or 40 mg. After 2 years, the median per cent change in carotid wall volume was +0.5% in the low-dose rosuvastatin group and −1.4% in the high-dose group. Subjects with carotid wall regression achieved lower mean LDL-C levels than those with atherosclerosis progression [1.78 vs. 2.17 mmol/l (69 vs. 84 mg/dl)]. Notably, rosuvastatin 5 and 40 mg reduced the size of the lipid-rich necrotic core by 18% and 36%, respectively, in the subset of subjects with such plaques at baseline, and nearly all patients showed regression from baseline. Moreover, no patients developed a new plaque with a lipid-rich necrotic core during treatment (57). Thus, treatment with rosuvastatin arrested progression of atherosclerosis and caused regression of the lipid-rich necrotic core in plaque.

Several studies have compared the effects of intensive vs. conventional statin therapy on coronary artery calcification measured by EBCT in subjects with subclinical atherosclerosis (58,59). Over a 1-year period, coronary artery calcification progressed similarly, regardless of whether subjects received a low or high dose of statin therapy and independently of the achieved serum LDL-C level. These findings suggest either that (i) atherosclerotic calcium deposition may not be reversible by statin therapy or (ii) a 12-month follow-up period may be too short to demonstrate a beneficial effect of statins on coronary artery calcification.

## Key questions and future directions

### Who should be screened, and which tests are appropriate?

As the technology for early diagnosis of atherosclerosis advances and becomes available for clinical use, a key question is who should be screened. In 2006, the American Heart Association Committee on Cardiovascular Imaging and Intervention issued a scientific statement on the clinical utility of detecting calcified plaque and the appropriate use of CT in a variety of clinical situations (60). A clinical expert consensus statement by the American College of Cardiology and the American Heart Association suggests that this relatively inexpensive technique may be appropriate for patients at intermediate risk based on their Framingham score, if there is a likelihood that a high coronary artery calcium score may reclassify these patients as higher risk and therefore requiring more intensive treatment (61). Although recommendations for the appropriate use of cardiovascular imaging in primary prevention are limited in number, many cardiologists today, in order not to underestimate risk, are using additional imaging modalities in asymptomatic patients with a strong family history of cardiovascular disease and/or a 10-year Framingham risk of ≥ 20%.

### Should patients with subclinical atherosclerosis be treated?

In several early studies, reductions in surrogate outcomes with lipid-lowering treatment appeared to correlate highly with reductions in cardiovascular events (49,62,63). However, although some trials have shown trends toward improved clinical outcomes following treatment for asymptomatic atherosclerosis (36,64), no imaging study to date has been designed or powered to prospectively correlate regression or lack of progression of atherosclerosis with reduction in risk for clinical events. The American College of Cardiology/American Heart Association consensus document on coronary artery calcium correlates pooled data for clinical outcomes with coronary artery calcium scores (61). The fact that persons with low coronary artery calcium scores have a very low risk for ‘hard’ CHD events (49 events/11,815, 0.4%) corroborates a correlation between atherosclerosis and clinical events. There is currently no position paper on carotid IMT, and we do not know the extent to which magnitude of change (increase, stabilisation or decrease) in carotid IMT correlates with disease outcomes.

The clinical significance of reducing the volume of atherosclerotic lesions using lipid-lowering treatment has not been established, and studies using surrogate end-points have not yet evaluated the magnitude of effects on clinical end-points. For example, we do not yet know how much plaque reduction would be required to lower cardiovascular risk by 10%, 20% or more. We do know that in a diffuse condition such as atherosclerosis, aggressive and comprehensive risk factor management provides equal efficacy in reducing risk for ACS compared with prophylactic percutaneous transluminal angioplasty with stenting (65). Therefore, if cardiovascular imaging establishes the presence of atherosclerotic disease in asymptomatic patients, many clinical trials support the use of intervention with statin therapy to reduce the rate of disease progression. Pending future randomised clinical trials designed to correlate reductions in atherosclerosis with clinical outcomes in persons without CHD but with evidence of subclinical atherosclerosis, the majority of clinicians would evaluate global risk factor burden in these individuals and treat them as high-risk patients in an effort to optimally impact the course of disease.

## Conclusions

Atherosclerosis is a chronic and progressive disease that causes substantial cardiovascular morbidity and mortality. Although often detected when patients first experience a major cardiovascular event, several techniques can be used to identify atherosclerosis when it is still in its subclinical stages. Reducing atherogenic lipoprotein burden with statin therapy is associated with significant changes in rates of coronary and carotid atherosclerosis disease progression. Some degree of plaque regression can be observed with aggressive statin therapy to lower LDL-C. As shown in the Pravastatin or Atorvastatin Evaluation and Infection Therapy – Thrombolysis in Myocardial Infarction 22 trial (66), such aggressive LDL-C lowering is also associated with significantly greater reductions in risk for acute cardiovascular events, need for revascularisation and hospitalisation for unstable angina pectoris compared with more moderate lipid lowering. Other recent clinical outcome trials (67,68) also underscore the considerable need for aggressive lipid lowering with statins in patients with established atherosclerotic disease.
